# Editorial for the Special Issue on Broadband Terahertz Devices and Communication Technologies, 2nd Edition

**DOI:** 10.3390/mi17060672

**Published:** 2026-05-29

**Authors:** Jiao Zhang, Lu Zhang, Kaihui Wang, Xiongbin Yu

**Affiliations:** 1 Purple Mountain Laboratories, Nanjing 211111, China; 2 College of Information Science and Electronic Engineering, Zhejiang University, Hangzhou 310027, China; 3 School of Information Science and Technology, Fudan University, Shanghai 200433, China; 4 Pengcheng Laboratory, Shenzhen 518000, China

The exponential growth of user data traffic imposes increasingly stringent requirements on communication networks regarding bandwidth, data rates, and diversified capabilities to support future social and industrial information exchange. Consequently, academic and industrial communities have actively pursued research and development (R&D) efforts to explore the feasibility of mobile broadband systems operating in frequency bands above 100 GHz. The terahertz band, spanning 0.1–10 THz and situated between microwave and optical frequencies, is regarded as a critical breakthrough for revolutionizing communication technologies, owing to its abundant spectrum resources [1,2,3]. As such, it has been widely recognized as a promising candidate for future bandwidth-intensive applications, particularly sixth-generation (6G) communications. In 2024, the ITU Radiocommunication Sector (ITU-R) approved a new report entitled “Technical Feasibility of IMT in Bands Above 100 GHz”, providing comprehensive technical information on frequency bands between 100 GHz and 400 GHz [4]. The terahertz band is capable of satisfying the ultra-high bandwidth and ultra-large capacity demands of emerging scenarios, thereby facilitating advanced services such as virtual reality, augmented reality, and satellite–terrestrial communications [5]. Furthermore, terahertz technology has also found promising applications in data center interconnection and integrated space–ground networks, reflecting its growing role in diverse high-performance communication infrastructures [6].

Building upon the success of the first edition of the Broadband Terahertz Devices and Communication Technologies Special Issue [7], this second edition continues to explore the state-of-the-art advances in the terahertz field. This Special Issue assembles 15 peer-reviewed articles, including 2 review papers and 13 original research articles. The covered research scope involves key broadband terahertz devices, system-level verification of terahertz communication systems, and innovative technologies at other low-frequency bands, which can also provide valuable insights and inspiration for subsequent terahertz research.

Five studies (Contribution 1–5) present power amplifiers, low-noise amplifiers and traveling wave tube amplifiers at different frequency bands, which could effectively expand the coverage range. Hu et al. (Contribution 1) and Shen et al. (Contribution 2) proposed a V-band high-gain wideband power amplifier (PA) and a compact V-band low-noise amplifier (LNA), respectively. The former PA prototype achieved a peak gain of 27.3 dB at 64 GHz, with a 3 dB bandwidth exceeding 13 GHz and a maximum saturated output power of 19.7 dBm using a 130 nm SiGe BiCMOS process. The latter LNA prototype exhibits a gain variation of less than 1.5 dB, a noise figure variation of less than 1.2 dB at test frequencies from 40 GHz to 65 GHz, and also shows excellent temperature robustness over a broad range from −55 °C to 85 °C. Yuan et al. (Contribution 3) designed two wideband high-efficiency power amplifiers operating in the X and Ku bands with peak saturated output powers of 31.2 dBm and 30.8 dBm, respectively, and fabricated using a 0.25 mm GaAs pseudomorphic high electron mobility transistor (pHEMT) process. Liu et al. (Contribution 4) proposed a dual-side hybrid embedding network to mitigate gain degradation in terahertz amplifiers at frequencies near *f*_max_; a four-stage amplifier is designed with a power gain of 19.3 dB at 280 GHz, corresponding to an 11.5 dB improvement over a conventional unboosted amplifier. Furthermore, Jamil et al. (Contribution 5) presented the design and optimization of a periodic cusped magnet-focused sheet beam optical system for application in a 340 GHz traveling wave tube amplifier.

Wang et al. (Contribution 6) presented a wideband D-band frequency sextupler chain in a 100 nm GaAs pHEMT process, comprising an input-stage tripler, an inter-stage harmonic-rejection power amplifier, and an output-stage doubler. The balanced tripler suppresses the fundamental, while the inter-stage PA enhances harmonic rejection and provides sufficient drive. The single-balanced doubler extracts the second harmonic while suppressing odd-order components. The prototype achieves 2.33 dBm peak output power with 0.33 dB conversion gain and an 18.9% fractional bandwidth (126.3–152.7 GHz). The 5th and 7th harmonics are suppressed by over 20 dBc, demonstrating suitability for wideband D-band signal generation.

Liu et al. (Contribution 7) proposed a dual-band bandpass filter with a wide upper stopband, integrating stepped-impedance resonators and a low-pass filter. The filter operates at 2.5 and 5.35 GHz with insertion losses of 0.12 dB and 0.6 dB, respectively, and achieves a wide upper stopband extending from 6.1 to 25 GHz to suppress high-frequency interference. Simulated and measured results show good agreement.

Electromagnetic metamaterials, engineered to manipulate wave properties including amplitude, phase, and wavefront, hold a prominent position in the field. Xu et al. (Contribution 8) developed a Metamaterial Incident Photon Reconstruction Theory (MIPRT), which reveals that metamaterials modulate incident photons through coherent destructive interference rather than absorption and re-emission. Validated in a single-layer metamaterial, MIPRT derives a unique resonant phase-amplitude relationship and offers a novel perspective on electromagnetic response mechanisms. Through scattering theory analysis of representative structures, Xu et al. (Contribution 9) derived and verified two explicit corollaries on transmission-reflection coupling, offering new insight into fundamental modulation mechanisms.

Terahertz detectors hold a considerable promise for applications spanning communications, imaging, spectroscopy, and sensing. Wang et al. (Contribution 10) designed a photonic terahertz antenna for the 275–296 GHz band, integrating modified uni-traveling carrier photodiodes (MUTC-PDs), impedance matching networks, a Wilkinson power combiner, and a Vivaldi antenna. Simulations show 1.58 dBm saturated output power at 280 GHz, a 4.13-fold enhancement over standalone devices via optimized impedance matching and power combining. The antenna achieves 7.93 dBi peak gain with reflections below −10 dB. Combining efficiency exceeds 95% under 20° phase imbalance, while the Wilkinson combiner shows only 0.76 dB insertion loss at 285 GHz. Tang et al. (Contribution 11) presented a 380 GHz zero-bias detector based on an ACST Schottky diode for terahertz radiometer receivers. A high-impedance grounding topology suppresses parasitic resonances and enhances stability, while a compact U-shaped waveguide transition realizes an inline configuration for simplified integration. Measurements demonstrate a peak voltage responsivity of 2318 V/W and a linearity of 0.9996 at 380 GHz, validating the proposed design for high-frequency radiometer and sensing applications.

Benefiting from broadband terahertz devices, terahertz communication systems have undergone rapid evolution in recent years. Gao et al. (Contribution 12) compared all-optical and all-electric receivers over 15 m and demonstrated HD video transmission over 200 m and 2 km using a 100 GHz all-optical transceiver. The all-optical transceiver achieves 11.318 Gbps error-free transmission over 200 m without clock recovery, whereas the all-electric receiver is limited to 3.125 Gbps over 15 m. These results validate the all-optical receiver for ultra-wideband, high-capacity 6G wireless communications.

Ensuring transmission security for high-speed terahertz wireless links remains a paramount concern in the field of terahertz research. Wang et al. (Contribution 13) proposed a secure, spectrum-efficient radio-over-fiber (RoF) system using compressive sensing (CS) and chaotic encryption. An 8 Gbit/s system is demonstrated over 10 km fiber and 20 GHz wireless links. Spectrum efficiency is enhanced by compressing data and reducing quantization bits. The system recovers signals with 6-bit fronthaul quantization, yielding SSIMs of 0.952 and 0.073 for legitimate (Bob) and unauthorized (Eve) receivers, respectively, at a 0.75 compression ratio, validating the proposed security.

Terahertz communication is pivotal for 6G, addressing data demands beyond current microwave and mmWave capabilities. However, Tbps kilometer-range transmission faces severe free-space path loss (>120 dB/km) and atmospheric absorption—the “dual attenuation dilemma.” Yu et al. (Contribution 14) summarized their advancements in long-distance terahertz communication through photonic–electronic co-design, including photonically assisted generation, polarization-multiplexed MIMO with MRC, high-gain antenna-lens arrays, and InP amplifiers. Experimental milestones include 1.0488 Tbps PS-64QAM (330–500 GHz), 30.2 km D-band transmission, 3 km fog-penetrating link at 312 GHz, and 100 Gbps satellite–terrestrial links beyond 36,000 km, validating THz viability for extreme-capacity 6G backhaul.

Finally, the multi-scenario applications of terahertz technology with free-space optics are reviewed. Liu et al. (Contribution 15) reviewed integrated terahertz and free-space optical (FSO) systems for future 6G and space–air–ground integrated networks (SAGIN). It examines advances in channel modeling, transmission performance, and integrated architectures, analyzing attenuation, turbulence, pointing errors, and noise under diverse atmospheric conditions. Terahertz achieves multi-Tbps rates over kilometers but suffers from molecular absorption and weather attenuation, whereas FSO offers superior bandwidth-distance products yet is vulnerable to turbulence-induced fading. Hybrid terahertz and FSO systems using adaptive switching enhance reliability and spectral efficiency, demonstrating Tbps-class data rates. Challenges remain in hardware integration and resource allocation, while future directions include unified channel models and intelligent switching for robust infrastructures.

The contributions presented in this Special Issue collectively advance terahertz devices and communication technologies, offering both profound theoretical insights and substantial engineering value. As Guest Editors, we extend our sincere gratitude to the authors for their outstanding contributions, to the reviewers for their rigorous and constructive feedback, and to the editorial team for their steadfast dedication throughout the publication process.
